# Structure and Bioactivities of a Novel Polysaccharide Extracted From *Dendrobium huoshanense* by Subcritical Water

**DOI:** 10.3389/fnut.2022.877871

**Published:** 2022-04-26

**Authors:** Li Wang, Yi-gui Mao, Xiang Zeng, Na Liu, Chao-fei Niu, Xin-xin Li, Bing-ji Ma, Lan-ping Guo, Xiao-long Yang

**Affiliations:** ^1^The Modernization Engineering Technology Research Center of Ethnic Minority Medicine of Hubei Province, School of Pharmaceutical Sciences, South-Central University for Nationalities, Wuhan, China; ^2^Department of Traditional Chinese Medicine, Henan Agricultural University, Zhengzhou, China; ^3^State Key Laboratory Breeding Base of Dao-di Herbs, National Resource Center for Chinese Materia Medical, China Academy of Chinese Medical Sciences, Beijing, China

**Keywords:** *Dendrobium huoshanense*, polysaccharide, subcritical water, structure, bioactivities

## Abstract

In this study, the polysaccharide was extracted by subcritical water from *Dendrobium huoshanense*. A novel polysaccharide (DHPs-1) was obtained through several purification steps and its structure and bioactivity were investigated. Structural analysis indicated that the weight-average molecular weight of DHPs-1 was 5.0 × 10^4^ Da and it was mainly composed of glucose (65.04%), mannose (14.23%), galactose (8.17%), galacturonic acid (6.41%), rhamnose (2.34%), and xylose (1.25%). 1,4-Glc*p*, and 1,4,6-Gal*p* were existed in the backbone of DHPs-1. The residues of 1,3,4-Gal*p*, 1,4-Man*p*, 1,4-Gal*p*, and 1,3,4,6-Gal*p* could be in the backbone or the side chains with the non-reducing terminal of α-Man*p*. Bioactivity tests indicated that DHPs-1 had immunomodulatory activity in that it significantly enhanced transcript levels of cytokines [Tumor necrosis factor-α (TNF-α), Interleukin-6 (IL-6), Interleukin-1β (IL-1β), and Interleukin-10 (IL-10)]. DPPH and hydroxyl radical scavenging tests showed that it had good antioxidant activity. These results reveal that DHPs-1 could be developed as a safe immunomodulatory agent and antioxidant for pharmacological or functional food applications.

## Introduction

Plants of the *Dendrobium* genus Orchidaceae are used as a tonic in many oriental countries for a long time (1). They have been used to make teas and soups; they can promote the secretion of body fluids, enhance the body's immunity, reduce fever, and prevent cardiovascular problems (2). According to the records of traditional Chinese medicine, among the *Dendrobium* species, *D. huoshanense, D. officinale, D. nobile*, and *D. chrysotoxum* are deemed superior and used widely as high-grade tonics both in traditional medicine and in the diet (1).

*Dendrobium huoshanense* contains kinds of chemical components, including polysaccharides, aromatic compounds and alkaloids. Among them, polysaccharides were reported to be the main component for the health benefits of *D. huoshanense*, namely its antioxidant antitumor and immunomodulatory activities (2). It is regarded that most of the polysaccharides' bioactivities are due to their ability to regulate to the innate immune system (3). Macrophages are considered ideal cell models to evaluate the immunomodulatory activities of polysaccharides (4). Polysaccharides can modulate the proliferation and activation of immunocompetent cells by enhancing the expression of cytokines. The polysaccharides' bioactivities are related with their monosaccharide composition, molecular weight and chain conformation, the extracted, isolated and purified methods (5).

The extraction method of polysaccharides affects the amount and types of polysaccharides recovered as well as the properties, structures and bioactivities of the polysaccharides. The extraction of polysaccharides from *D. huoshanense* is usually accomplished using a conventional extraction technique with water or aqueous organic solvents. However, this method requires long extraction time and has low extraction efficiency. The subcritical water extraction method is a relatively new method that is recognized as more rapid and efficient for extracting active ingredients from plant materials than conventional extraction methods. Furthermore, subcritical water extraction is considered to be environmentally friendly and safe as water is the only extraction solvent (6, 7).

In this study, a novel polysaccharide was extracted and purified from *D. huoshanense*, named DHPs-1, and its structure was determined by HPAEC, GC-MS, GPC, FT-IR and NMR spectroscopy. Then, the *in vitro* antioxidant activities of DHPs-1 were evaluated. The cytotoxicity of DHPs-1 was evaluated using the CCK-8 method. Additionally, the transcript levels of cytokine genes in RAW264.7 cells after the purified polysaccharide administration was also identified using qRT-PCR.

## Materials and Methods

### Materials and Reagents

Wild plants of *D. huoshanense* growing in Huoshan County, Anhui Province, China, were collected. Dimethyl sulfoxide (DMSO), acetonitrile, sodium hydride (NaH), and iodomethane (CH_3_I) lipopolysaccharide (LPS) were purchased from Sigma Chemical Co., USA. DEAE-cellulose and Sephadex G-100 were purchased from Solarbo Co., Ltd. (Beijing, China). The Cell counting kit-8 (CCk-8) was purchased from Han Heng Biotechnology Co., Ltd. (Shanghai, China). The ascorbic acid (VC) was purchased from Sinopharm Chemical Reagent Co., Ltd. (Shanghai, China). All the other reagents or chemicals were of analytical grade.

### Extraction and Purification

The stems were air-dried, ground to powder and refluxed with 95% ethanol (1:200, g/mL) for three times, each time for 5 h. This process was done to remove the small molecular compounds. A Synthware® pressure glass vial (Beijing Synthware Glass, Inc., China) with a volume of 350 mL was used for the *D. huoshanense* polysaccharide extraction by HCW, which is water under subcritical pressure and temperature. The air-dried powder of *D. huoshanense* (5 g) and water (100 mL) were added into the reactor, and the reactor was sealed. The mixture was heated up to 150°C (0.4757 Mpa). The sample was extracted for 180 min and each sample was extracted in this way two times. After centrifugation, the supernatants from the two extractions were combined and concentrated, and then precipitated by adding 4 times the volume of anhydrous alcohol. The precipitate was collected by centrifugation, redissolved in water and deproteinated by the Sevage method (polysaccharide solutions:chloroform:isopentanol = 25:4:1, v/v/v) 6 times. The deproteinized polysaccharides were then decolorized by HP20 macroporous resin at 25°C for 4 h (120 r/min). The supernatant was combined, concentrated and dialyzed using 3,500 Da, and finally lyophilized. The yield of crude polysaccharides was calculated by the following equation:


$$\begin{array}{l} yield\;\left(\%\right)=\frac{\text{Crude polysaccharide weight }\left(g\right)}{\text{Raw material weight }\left(g\right)}\times 100\% \end{array}$$

The crude polysaccharide was further purified using DEAE-cellulose and Sephadex G-100. Then, the eluents were collected and determined using the phenolsulfuric acid method using α-glucose as a standard, and elution curves were drawn. Finally, white (DHPs-1) purified polysaccharides was obtained. The yields of DHPs-1 were calculated by the following equation:


$$\begin{array}{l} yield\;\left(\%\right)=\frac{DHPs-1\left(g\right)}{\text{Crude polysaccharide weight }\left(g\right)}\times 100\% \end{array}$$

### Characterization

The total carbohydrate content, uronic acid content and protein content was determined by phenol-sulfuric acid method using D-glucose as a standard, mhydroxydiphenyl method and using galacturonic acid as a standard and Coomassie brilliant blue method with bovine serum albumin as a standard, respectively (8).

#### Monosaccharide Composition and Molecular Weight Analyses

According to our previous methods, the monosaccharide composition and molecular weight of DHPs-1 were analyzed by high performance anion exchange chromatography (HPAEC) and gel permeation chromatography (GPC), respectively (7, 9).

#### FT-IR and NMR Spectroscopy

According to our previous methods, the IR spectra and 1D and 2D NMR spectra of DHPs-1 were analyzed by a Nicolet FT-IR spectrometer (Thermoscientific, USA) and a Bruker ARX500 spectrometer, respectively (7, 9).

#### Glycosidic Linkage Analysis

DHPs-1 was methylated according to Wang et al. (7). The sample was methylated, hydrolysed, reduced and acetylated. The partially methylated alditol acetates (PMAAs) were determined by a gas chromatography/mass (GC/MS) system (Agilent 6890/5975, USA). Briefly, 5 mg of polysaccharide was dissolved in DMSO/NaOH under nitrogen and then methylated with CH_3_I. The fully methylated sample was hydrolyzed with 2.5 mol/L TFA at 121°C for 1.5 h. After reduction and acetylation of the hydrolysates, the partially methylated alditol acetate (PMAA) derivatives were analyzed with a GCMS system (Agilent 7890A/5975C, USA) fitted with a HP-5 ms quartz capillary colum (30 m × 0.25 μm × 0.25 mm).

#### Molecular Morphology Analysis

The molecular morphology of DHPs-1 was observed using a scanning electronic microscope (SEM) (FEI Quanta 250 FEG). The sample was sputtered-coated with platinum using a Cressington 108auto Sputter Coater, and the images were obtained at a voltage of 3.0 kV with 800- and 1,000-fold magnification under high vacuum.

### Bioactive Analyses

The antioxidant activities of DHPs-1 had been determined using Vc as the positive control, and the macrophage proliferation and Q-PCR assays were determined using LPS as positive controls.

#### Antioxidant Assays

##### DPPH Radical Scavenging Assay

The 1,1-Diphenyl-2-picrylhydrazy (DPPH) radical-scavenging activity and hydroxyl radical scavenging activity of DHPs-1 were assessed according to the method of Wang et al. (7). Briefly, 1 mL of each sample solution with specific concentration (0.05, 0.1, 0.5, 1, 2, 4, 6, 8, and 10 mg/mL) was mixed with 1 mL of 0.05 mmol/L DPPH solution, respectively. For positive control, vitamin C (Vc) was used as antioxidant reactant. After dark reaction for 30 min, the absorbance of each mixture was measured at 517 nm with a microplate reader. The DPPH radical scavenging rate was calculated by the following formula:


$$\begin{array}{l} \text{DPPH radical scavenging rate }\left(\%\right)=\;\left[1-\frac{A_{X}-A_{X0}}{A_{0}}\right]\times 100 \end{array}$$

*A*_0_ is the absorbance of the mixture without sample,

*A*_*X*_ is the absorbance of sample solution group,

*A*_*X*0_ is the absorbance of the mixture without the DPPH solution.

##### Hydroxyl Radical Scavenging Assay

The concentrations used for each sample and reaction carrier are the same as described above in 2.4.1.1 For measurement, 50 μL of each sample solution was sequentially mixed with equivalent volume FeSO_4_ solution (4.5 × 10^−3^ moL/L) and salicylic acid ethanol solution (4.5 × 10^−3^ mol/L). After mixing, 100 μL of 6 × 10^−3^ mol/L H_2_O_2_ was added, and kept for 37°C, 30 min. Vc was used as the positive control. Absorbance of the reaction solutions at 510 nm was recorded to calculate the hydroxyl radicals scavenging rates for each polysaccharide sample, using the following formula:


$$\begin{array}{l} \text{Hydroxyl radical scavenging rate }\left(\%\right)=\;\left[1-\frac{A_{X}-A_{X0}}{A_{0}}\right]\times 100 \end{array}$$

*A*_0_ is the absorbance of the mixture without sample,

*A*_*X*_ is the absorbance of the sample solution group,

*A*_*X*0_ is the absorbance of the mixture without the H_2_O_2_ solution.

#### Cell Viability Analysis

The cell viability was determined according to the method reported by Zhang et al. (10). RAW264.7 cells were cultured in 96-well plates for 24 h and then treated with DHPs-1 at different concentrations (5, 10, 20, 40, 80, 160, 320, 640, and 800 μg/mL) or lipopolysaccharide (LPS, as the positive group, 1 μg/mL) for 24 h after the medium was aspirated. Ten microliter CCK-8 regent was added to each well, and the mixture was measured after reaction for 1 h with Varioskan Flash System Enzyme Labeler (Thermo Scientific, USA) at 450 nm. With only CCK-8 mixture was considered as the blank group. The proliferation rate of RAW264.7 was measured using the following formula:


$$\begin{array}{l} \text{Proliferation rate}\left(\%\right)\\ \;=\;\left[{\text{OD}}_{\text{DHPs}-\text{1}}-{\text{OD}}_{\text{Blank}}\right)/({\text{OD}}_{\text{Control}}{\text{OD}}_{\text{Blank}})]\\ \;\times \;100 \end{array}$$

#### RT-PCR Analysis

The mRNA transcriptions of TNF-α, IL-1β, IL-6, IL-10 genes induced by DPHs-1 or LPS groups were determined according to the method reported by Zhang et al. (10). Briefly, The RAW264.7 cells were cultured with DHPs-1 (20, 40, 80 μg/ml) at 37°C for 24 h, using LPS as a standard. TNF-α, IL-6 IL-10, and IL-1β were determined by the level of mRNA was expressed with GAPDH as the internal reference. The primers for above four cytokines were designed and listed in Table 1.

**Table 1:** Sequences and amplification conditions for RT-PCR primers.

**Primer**	**Sequences**	**Annealing temperature (°C)**
GAPDH	F5′-ACCCCAGCAAGGACACTGAGCAAG-3′ R5′-GGCCCCTCCTGTTATTATGGGGGT-3′	60
TNF-a	F5′-GGAAAGGACACCATGAGC-3′ R5′-CCACGATCAGGAAGGAGA-3′	64
IL-1β	F5′-CCGCAGCCTACATCATTC-3′ R5′- CGCCATAAGCCCTCCTA-3′	63
IL-6	F5′- TGTGTGAAAGCAGCAAAGA-3′ R5′- ACCAGGCAAGTCTCCTCA-3′	62.5
IL-10	F5′- GCCCTTTGCTATGGTGTC-3′ R5′- TCTCCCTGGTTTCTCTTCC-3′	63.1

### Statistical Analysis

All experiments were tested in triplicate. The data were analyzed using IBM SPSS Statistics 20.0, and mean comparisons were carried out using Duncan's multiple range test. A value of *p* < 0.05 was considered to be statistically significant.

## Results and Discussion

### Isolation, Purification, Physicochemical Composition, and Molecular Weight Analyses of DHPs-1

**Figure 1:**
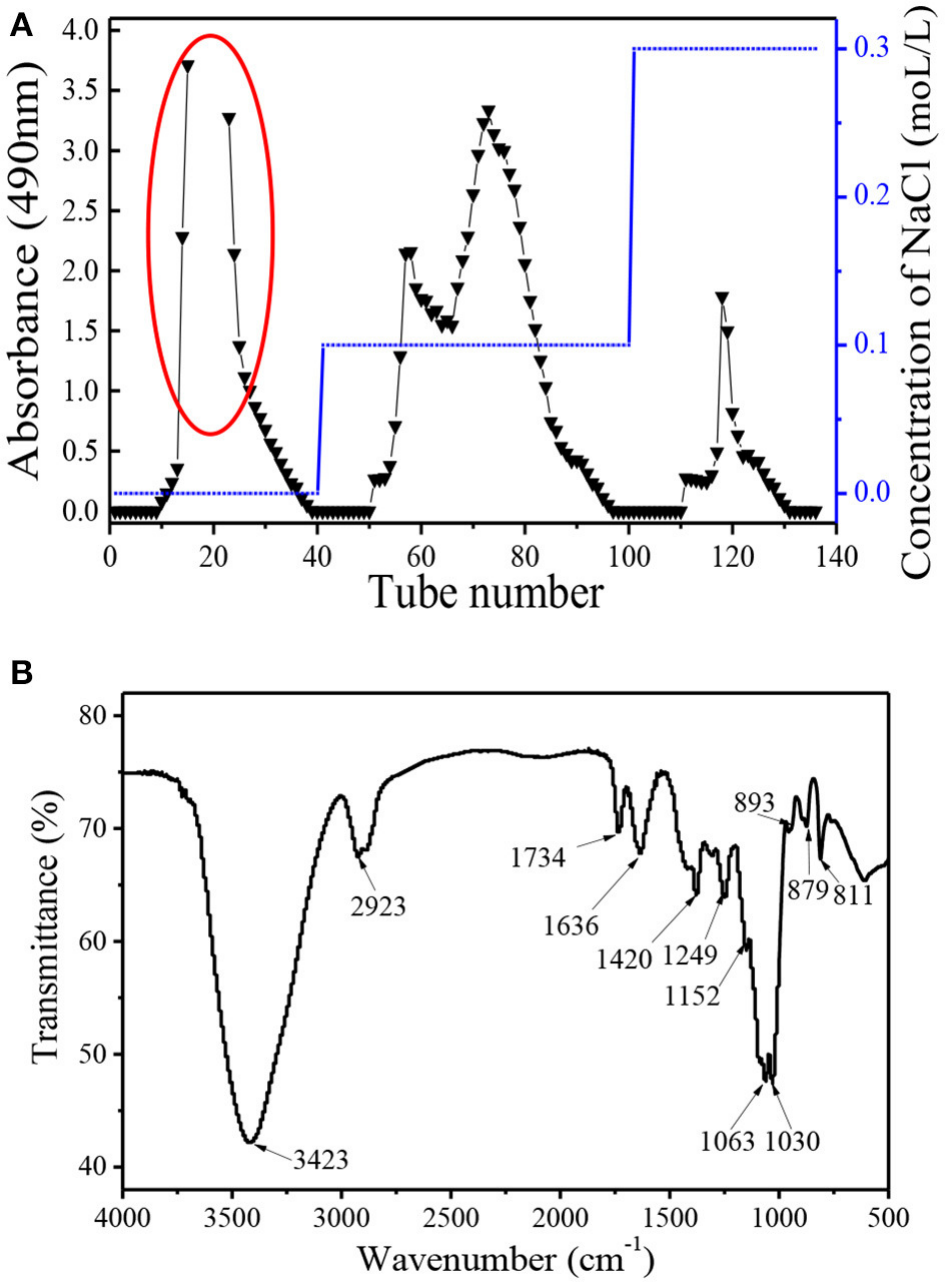
Elution curve of DHPs-1 on DEAE-52 **(A)**; FT-IR spectrum of DHPs-1 **(B)**.

*Dendrobium huoshanense* polysaccharide (DHP) was obtained using subcritical water extraction with a yield of 19.52%, which is the ratio of the crude polysaccharide to the raw material. The crude polysaccharide was deproteined and decolourized, and then further purified by the DEAE-52 column and Sephadex G-100 chromatography column. As shown in Figure 1, purification on the DEAE-52 column yielded four fractions: one deionized water fraction, two 0.1 moL/L NaCl fractions, and one 0.3 moL/L NaCl fraction. Their yields were: 21.5, 1.9, 6.0, and 2.4%, respectively. Due to the high yield of the deionized water fraction, it was further purified using a Sephadex G-100 chromatography column, and yielded a single fraction with a symmetrical peak with a yield of 89.3% (not shown); this fraction was named DHPs-1. The calculated weight-average molecular weight (Mw) and number-average molecular weight (Mn) of DHPs-1 were 50 and 36 KDa, respectively (Table 2). The polymer dispersity index (PDI) of DHPs-1 was about 1.39; the closeness to 1 suggests DHPs-1 was homogeneous. This result is different from the polysaccharides obtained from *Dendrobium huoshanense* (6.7 KDa, 232 KDa-8090 KDa) using the traditional extraction method (11–14). XRD analysis is often used to detect the crystalline or amorphous structure of the polysaccharides. The XRD image of DHPs-1 showed a strong diffraction peak at 2θ of 20°, and this result indicates that there was not only crystalline structure but also amorphous structure in DHPs-1 (see Supplementary Material). In fact, DHPs-1 had more amorphous than crystalline with the crystallinity degree of 18.5%. Actually, this crystal structure is directly determined by the flexibility, solubility, swelling and other properties of polysaccharides (15). This result suggests the DHPs-1 is a novel polysaccharide, and the reason was attributed to the different species, specific extraction and processing methods.

**Table 2:** Physicochemical properties of DHP.

	**DHP**
Yield (%)	19.5 ± 1.72
Carbohydrate (%)	97.8 ± 0.07
Protein (%)	2.7 ± 0.07
Uronic acid (%)	4.5 ± 0.30
Degree of crystallinity (%)	18.5 ± 0.25
Molecular weight (KDa)	
Weight-average molecular weight (Mw)	50.4 ± 4.07
Number-average molecular weight (Mn)	36.2 ± 4.07
Polymer dispersity index (PDI)	1.39

### FT-IR Spectrum Analysis

The FT-IR spectrum of DHPs-1 showed two characteristic peaks: a strong one at around 3,423 cm^−1^ representing the intense stretching vibration of O-H bonds, and a weak one at around 2,923 cm^−1^ representing weak C-H bonds (Figure 1B). The areas at 1,734 and 1,636 cm^−1^ indicated the presence of -COOR and -COOH, respectively, which indicates the existence of galacturonic acid or glucuronic acid and acetyl groups (15). The band between 950 and 1,200 cm^−1^ is often called the “fingerprint” of molecules, because the position and intensity of the bands are specific for different polysaccharide. The bands at 1,636 and 1,249 cm^−1^ revealed the presence of little amount of protein, which is consistent with above result (16). This result is in accordance with the result of the composition analysis shown in Table 1. The signals at 1,200–1,000 cm^−1^ indicated that DHPs-1 contained pyranose rings, which was attributed to C-O-C and C-O-H glycosidic bands (17). The band at 1,063 cm^−1^ indicated that the glucosidic linkages of the glucosyl residues were β-linked due to O-substituted glucose residues (18). In addition, the peaks at 890 and 811 cm^−1^ might be due to the presence of manopyranosyl and α-galactopyranosyl. In addition, the peak at 890–900 cm^−1^ was indicative of the presence of β-glycosidic linkages and the absorption peak at 853 cm^−1^ indicated the presence of α-glycoside bonds (7).

### Monosaccharide Composition and Methylation Analyses

DHPs-1 is mainly composed of glucose, mannose, galactose, galacturonic acid, rhamnose with the ratio of 65.04:14.23:8.17:6.41:2.34 (Table 3). There are also traces of xylose, arabinose, glucuronic acid, fructose, and D-glucosamine hydrochloride with the ratio of 1.25:0.95:0.61:0.24:0.24. Other studies have reported the component monosaccharides of *D. huoshanense* polysaccharides as follows: DHPD2 was mainly composed of galactose, glucose and arabinose with a molar ratio of 0.90:0.72:0.20 and traces of mannose, rhamnose and xylose (12); DHPIA was mainly composed of mannose, glucose and galactose with a molar ratio of 2.5:16.0:1.0 (11); TC-DHPA4 was composed of arabinose, rhamnose, mannose, glucose, galactose and glucuronic acid in a molar ratio of 1.00:1.28:1.67:4.71:10.43:1.42 (14); cDHPS was composed of d-mannose and d-glucose in a 3.04:1.00 molar ratio (13); DHP-4A was composed of glucose, mannose, arabinose and rhamnose in a molar ratio of 13.80:6.10:3.00:2.10 (19); HPS-1B23 was composed of glucose, mannose and galactose in a 31.00:10.00:8.00 molar ratio (20). The differences of molar ratio and the monosaccharide types are presumably due to different source material, and different extraction and purification methods.

**Table 3:** Monosaccharide composition of DHPs-1.

**Monosaccharide composition**	**Molar ratio (%)**
Arabinose	0.95
Glucose	65.04
Galactose	8.17
Xylose	1.25
Mannose	14.23
Fructose	0.24
Rhamnose	2.34
Galacturonic acid	6.41
Glucuronic acid	0.61
D-Glucosamine Hydrochloride	0.24

Methylation is a powerful tool to detect the polysaccharide's structure. Due to the low content of uronic acid, all the free hydroxyl groups of DHPs-1 are directly and completely methylated. The methylated DHPs-1 was hydrolysed, reduced and acetylated to obtain the PMAAs, and then were detected by GC-MS. The GC-MS result indicated that DHPs-1 has seven types of sugar linkages: Man*p*-(1→, → 4)-Man*p*-(1→, → 4)-Gal*p*-(1→, → 4)-Glc*p*-(1→, → 3, 4)-Gal*p*-(1→, → 4, 6)-Gal*p*-(1→, → 3, 4, 6)-Gal*p*-(1→ at molar percent ratios of 12.1:2.1:1.3:69.3:4.8:9.3:1.1, respectively (Table 4). Both results of monosaccharide composition and methylation linkage analysis, glucose was the main component of DHPs-1, which indicated that 1,4-Glc*p* residues exist in the backbone of DHPs-1. These molar percent ratios of different types of sugar residues were consistent with the percentages of sugars obtained above, except for galactose. This reason could be that the galacturonic acid peak overlapped with the galactose peak, causing an apparent increase of galactose content in the methylation result. The linkages of galacturonic acid could be the 3,4-Gal*p*A or 4,6-Gal*p*A. The number of terminal Man*p* residues approximately equaled the number of branched residues, indicating that the methylation method of DHPs-1 was effective. The terminal mannose was directly or indirectly attached to → 3, 4)-Gal*p*-(1→, → 4, 6)-Gal*p*-(1→, and → 3, 4, 6)-Gal*p*-(1→, as the ratio between them was close to 1:1.

**Table 4:** Methylation analysis for DHPs-1.

**Linkages**	**Derivative names**	**Retention Time**	**Molar ratio**	**Relative molar (%)**
t-Man(p)	1,5-di-O-acetyl-2,3,4,6-tetra-O-methyl mannitol	9.303	7.6	12.1
4-Man(p)	1,4,5-tri-O-acetyl-2,3,6-tri-O-methyl mannitol	13.361	1.3	2.1
4-Gal(p)	1,4,5-tri-O-acetyl-2,3,6-tri-O-methyl galactitol	14.158	0.8	1.3
4-Glc(p)	1,4,5-tri-O-acetyl-2,3,6-tri-O-methyl glucitol	14.495	43.5	69.3
3,4-Gal(p)	1,3,4,5-tetra-O-acetyl-2,6-di-O-methyl galactitol	16.699	3.0	4.8
4,6-Gal(p)	1,4,5,6-tetra-O-acetyl-2,3-di-O-methyl galactitol	18.798	5.9	9.3
3,4,6-Gal(p)	1,3,4,5,6-penta-O-acetyl-2-O-methyl galactitol	21.235	0.7	1.1

### NMR Spectral Analysis

**Figure 2:**
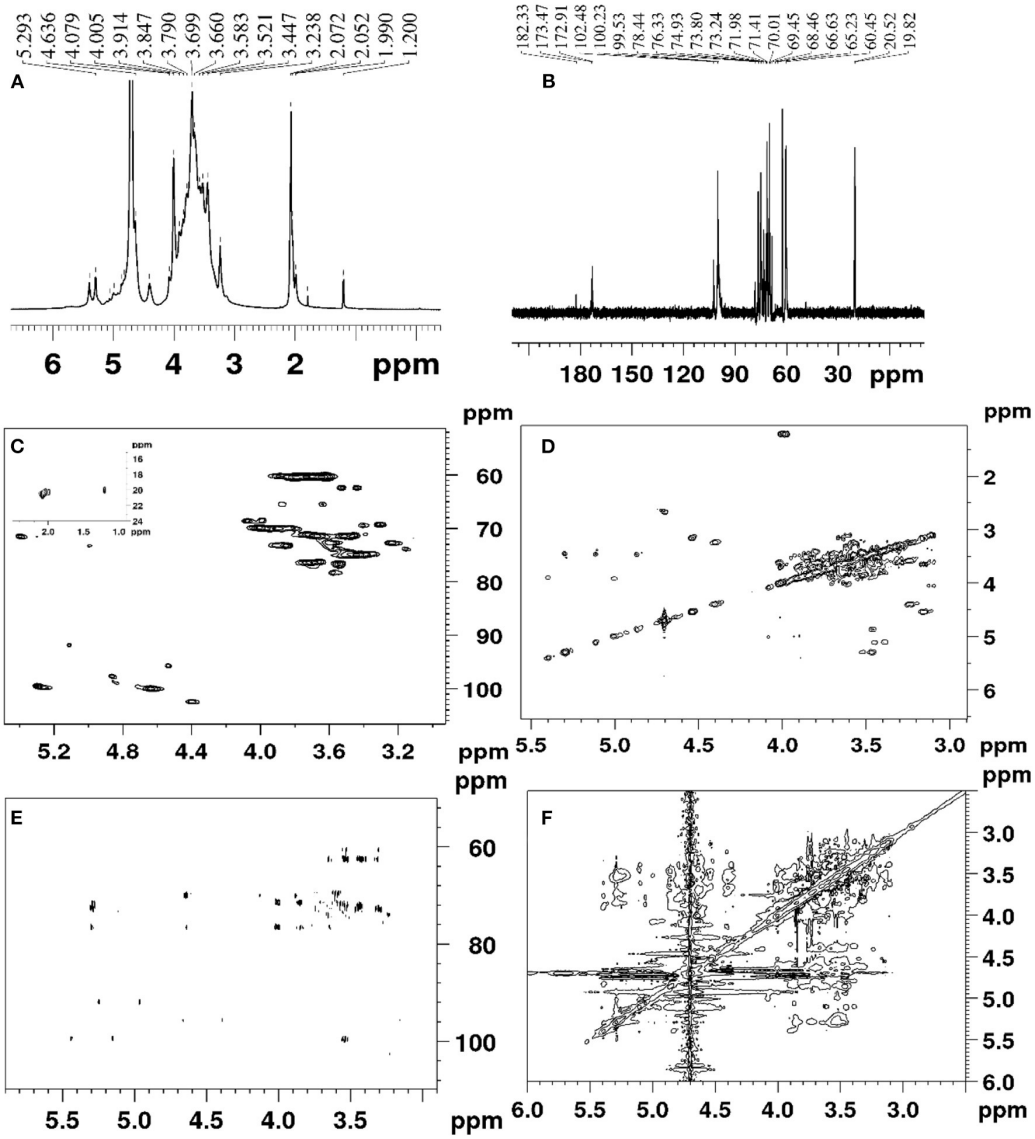
The spectra of DHPs-1: **(A)** ^1^H; **(B)** ^13^C; **(C)** HSQC; **(D)** COSY; **(E)** HMBC; **(F)** ROESY.

The DHPs-1 structure was further determined by 1D NMR and 2D NMR. The ^1^H, ^13^C, DEPT-135, HSQC, HMBC COSY ROESY spectra of DHPs-1 are shown in Figure 2. Three anomeric signals were observed in the region of 4.40–5.40 ppm for the ^1^H NMR (Figure 2A), suggesting that DHPs-1 was mainly composed of three sugar residues, and the glycosidic linkages contain β- and α-type. In the ^13^C NMR spectrum (Figure 2B), three anomeric carbon signals were observed at 100.08, 99.70, and 102.4 ppm, and the remaining signals appeared in the region of 60.21–78.42 ppm. The three sugar residues were designated as A, B, and C, respectively. According to the HSQC spectrum (Figure 2C), the anomeric carbon signals at A (δ 100.08), B (δ 99.70), and C (102.40 ppm) correlated to anomeric proton signals of δ 4.63, 5.27, and 4.39 ppm, respectively. The chemical shifts in the ^13^C spectrum and the DEPT-135 spectrum were similar. However, the CH_3_ and CH signals were positive, and the CH_2_ signals were negative in the DEPT-135 spectrum. According to the DEPT-135 spectrum (see Supplementary Material), C-6 signal peaks were mainly observed in the region of δ 60–70 ppm. Three main C-6 signal peaks, including δ 60.50, 62.36, and 60.21 ppm, were obtained (21). The anomeric signal (100.02 ppm) of 1,4-β-D-Man*p* was overlapped with the signal of 1,4-α-D-Glc*p*. No signal existed in the region of 82.0–88.0 ppm, indicating that all the sugar residues were of pyranose configuration. According to the literatures, 19.0–20.7 ppm and 172.6–174.1 ppm were investigated to be Me and CO of O-acetyl groups. This was also supported by the chemical shifts existed at 2.00–2.07 ppm in the ^1^H NMR spectrum of DPHs-1 (20). This result is in accordance with the results of monosaccharide composition and FT-IR analysis.

According to the COSY spectrum (Figure 2D), the chemical shifts observed at 4.05, 3.61 3.54, 3.66, and 3.56 ppm were assigned to H2, H3, H4, H5, and H6 of residue A, respectively. Taking into consideration of the ^13^C NMR and HSQC spectra, the corresponding ^13^C shifts can be obtained at 72.92, 78.24, 72.71, 76.15, and 60.50 ppm. Above all, residue A was the 1,4-β-d-Glc*p* (21, 22). The similar way can be used to assign the proton and carbon signals for residue B and residue C in 1D and 2D NMR spectra. Thus, the residues were designated as terminal-α-Man*p* (B), and 1,4,6-β-D-Gal*p* (C), respectively. The main chemical shifts of DHPs-1 are presented in Table 5.

**Table 5:** ^1^H and ^13^C NMR chemical shifts of DHPs-1 recorded in D_2_O.

**Sugar**	**Residues**		**1**	**2**	**3**	**4**	**5**	**6**
A	→ 4)-β-d-Glc*p*-(1→	C	100.08	72.92	78.24	72.71	76.15	60.50
		H	4.63	4.05	3.61	3.54	3.66	3.56
B	α-Man*p*-(1→	C	99.70	76.64	69.77	76.50	71.7	62.36
		H	5.27	3.55	3.86	3.48	3.70	3.44
C	→ 4,6)-β-Gal*p*-(1→	C	102.4	72.6	73.7	69.84	71.65	60.21
		H	4.39	3.25	3.58	3.26	3.65	3.81

The sequence of sugar residues was determined using HMBC, and the backbone structure of DHPs-1 was analyzed using HMBC spectrum. In the HMBC spectrum (Figure 2E), the chemical signals of DHPs-1 were assigned by a combination of the 1D and 2D NMR spectra. For example, the cross signal at 3.54/100.08 ppm revealed that the H-4 of → 4)- β-D-Glc*p*-(1→ and the C-1 of → 4)-β-D-Glc*p*-(1→ were connected, indicating the presence of → 4)-β-D-Glc*p*-(1→ 4)-β-D-Glc*p*-(1→. The cross signal at 4.63/69.84 ppm revealed that the H-1 of → 4)-β-D-Glc*p*-(1→ and the C-4 of → 4, 6)-β-D-Gal*p*-(1 were connected, indicating the presence of → 4)-β-D-Glc*p*-(1→ 4, 6)-β-D-Gal*p*-(1→. The cross signal at 4.63/76.5 ppm revealed that the H-1 of → 4)-β-D-Glc*p*-(1→ and the C-4 of α-D-Man*p*-(1 were connected, suggesting the presence of α-D-Man*p*-(1→ 4)-β-D-Glc*p*-(1→ (23–25). The ROESY spectrum of DPHs-1 (Figure 2F) shows cross-peaks: H1 of 1,4-β-D-Glc*p* with H2, H3, H5 at 4.63/4.05, 3.58, and 3.66 ppm, respectively; H1 of terminal-Man*p* with H4 at 5.27/3.84 ppm, revealing the presence of sugar residues: … → 4)-β-D-Glc*p*-(1→ 4)-β-D-Glc*p*-(1→ … and α-D-Man*p* → 4)-β-D-Glc*p*-(1→.

### SEM of DHPs-1

**Figure 3:**
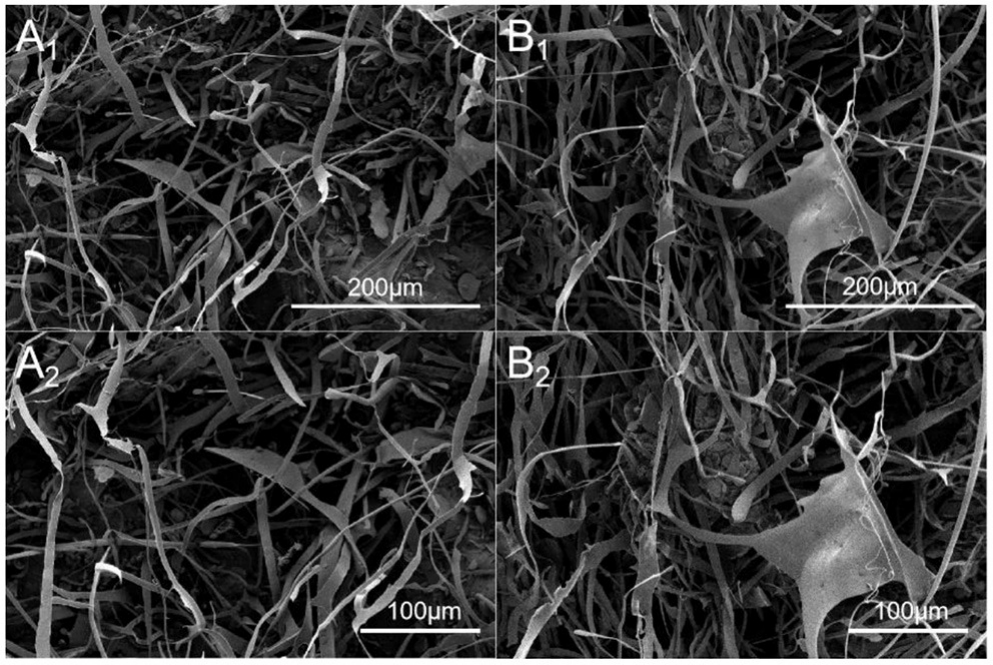
SEM images of DHPs-1 (**A**_**1**_**, A**_**2**_: different magnification of the same region; **B1, B2**: different magnification of the same region; **A**_**1**_**, B**_**1**_, 800×; **A**_**2**_ **B**_**2**_, 1,000×).

SEM was used to characterize the morphology of DHPs-1. Under high magnification (800× and 1,000×), it appeared as fibrous filaments and ribbons with branches (Figure 3). These microstructural characteristics may be attributed to the structure disruption of the cell wall and the disaggregation of the polysaccharide aggregates by the subcritical water extraction process, leading to the loosened texture of DHPs-1. The morphology of DHPs-1 was mainly affected by the subcritical water extraction method and the purification step, which could change the hydrogen bonds of polysaccharide molecule. The surface morphology and microstructure of DHPs-1 might be correlated with its physicochemical properties and antioxidant activities (26).

### Thermal Property of DHPs-1

**Figure 4:**
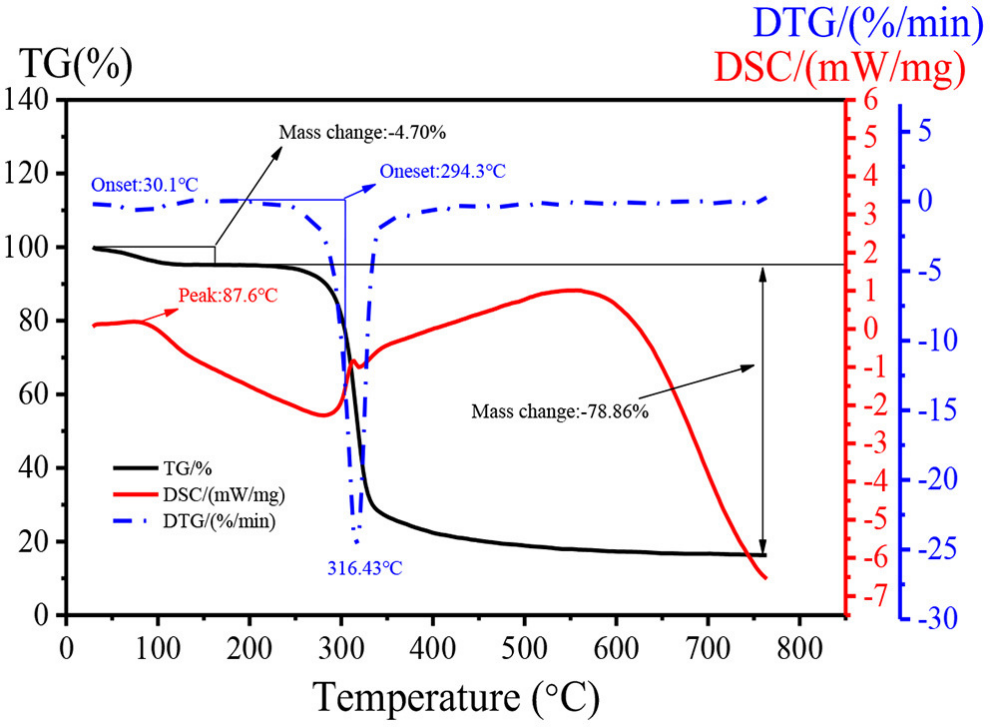
TG, DTG, and DSC curves of DHPs-1.

Changes in thermal stability can quantitatively reflect changes of material quality in the process of dehydration, oxidation, and decomposition. The thermal property of DHPs-1 was investigated using TG, DTG, and DSC analyses. Two stages of weight losses were obtained from the TG experiment over a wide temperature range (25–760°C) (Figure 4). The first weight loss occurred below 150°C with 4.70% of the original weight, which might be attributed to the loss of bounding water. The second stage of weight loss was observed at 200–350°C. The weight of DHPs-1 began to decrease sharply, and this could be attribute to the depolymerization and decomposition of DHPs-1. The weight loss in this region was 78.86% of the original weight. The maximum decomposition rate of DHPs-1 seen in the DTG curve occurred at 316.43°C. Simultaneously, the DSC curve was obtained. As shown in Figure 4, the DHPs-1 was characterized by one peak at 87.6°C corresponding to the glass transition temperature. One exothermic peak at 200–350°C was detected, which might be attributed to the chemical decomposition of DHPs-1 (27). Taking into consideration of TG, DTG, and DSC curves, DHPs-1 was relatively stable below 300°C. This result indicated that the thermal stability of DHPs-1 was higher than that of polysaccharides from Folium Isatidis (25). Consequently, there are differences in their thermal stabilities and these differences may be attributed to the different source material, extraction method, purification method, spatial conformation, monosaccharide compositions, or structures of polysaccharides.

### DPPH and Hydroxyl Radicals Scavenging Activities

**Figure 5:**
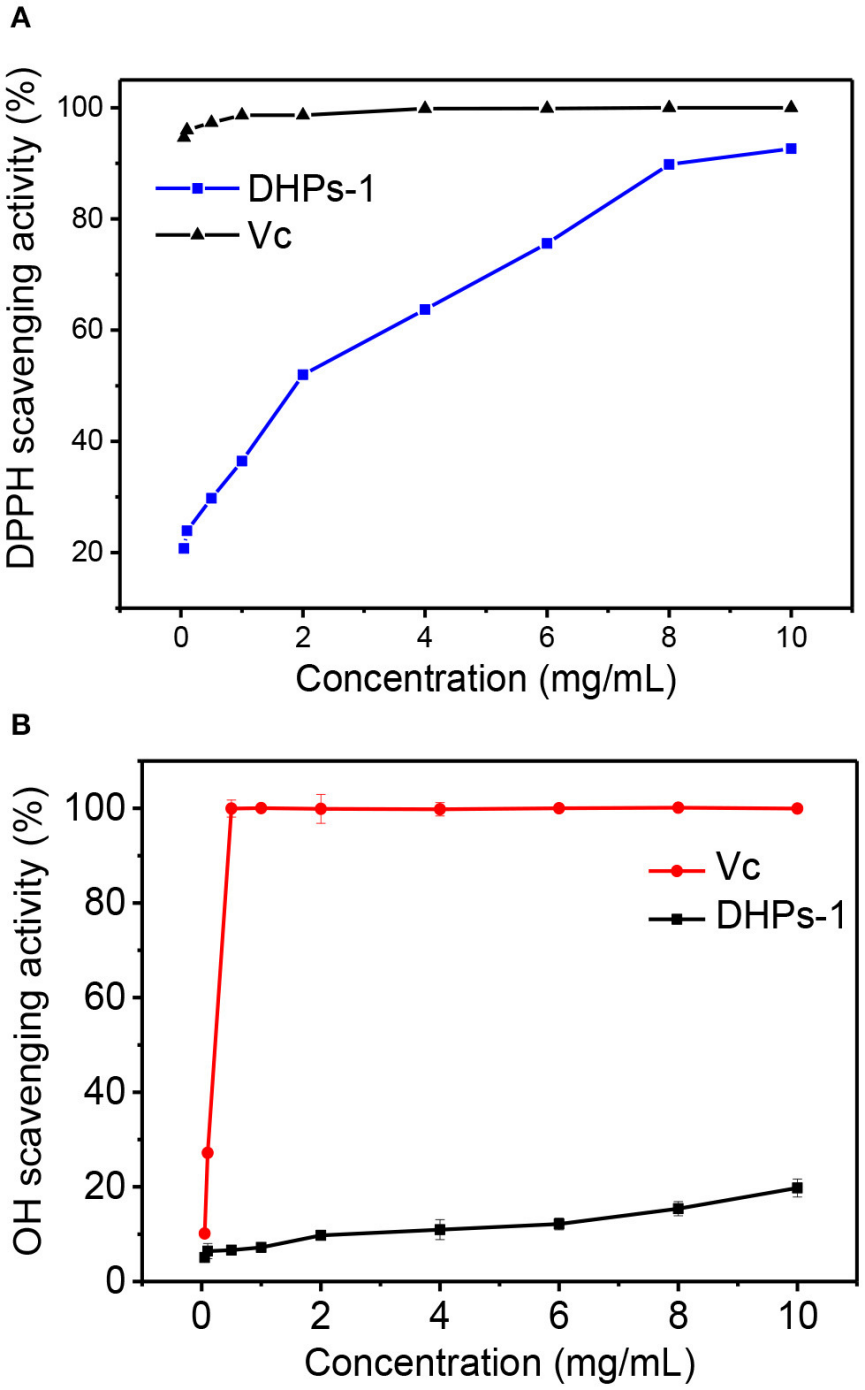
Antioxidant activity of DPHs-1. **(A)** DPPH radical scavenging assay; **(B)** Hydroxyl radical scavenging activity.

The DPPH· and ·OH scavenging activities of DHPs-1 are presented in Figure 5. The scavenging activity of DHPs-1 increased with the increase of polysaccharide concentration (0.05–10 mg/mL) (Figure 5A). At a concentration of 10 mg/mL, the DPPH· scavenging activity was 92.64%, which was close to that of Vc. The *Dendrobium huoshanense* polysaccharide was extracted twice with distilled water at 65°C and its scavenging rates of DPPH were increased from 31.4 to 38.7% with increasing the polysaccharide concentration from 0.5 to 2.0 mg/mL (11). The DPPH· scavenging activity of DHPs-1 was better than that of *Dendrobium huoshanense* polysaccharide obtained by traditional water extraction method. This might be attributed the different monosaccharide composition, molecular weight, chain conformation of polysaccharides obtained by different extracted and purified methods. The scavenging activity for hydroxyl radicals of DHPs-1 was in a concentration-dependent manner. The hydroxyl radical scavenging capacity of DHPs-1 (0.05–10 mg/mL) increased from 5.06 to 19.76% at concentrations between 0.05 and 10 mg/mL. With this level of antioxidant activity, DHPs-1 could be developed and used in natural medicine and functional food production (28). However, the antioxidant mechanism of DHPs-1 is unclear at present, and need to be investigated further.

### Immunomodulatory Activity of DHPs-1

#### Macrophages Proliferation

**Figure 6:**
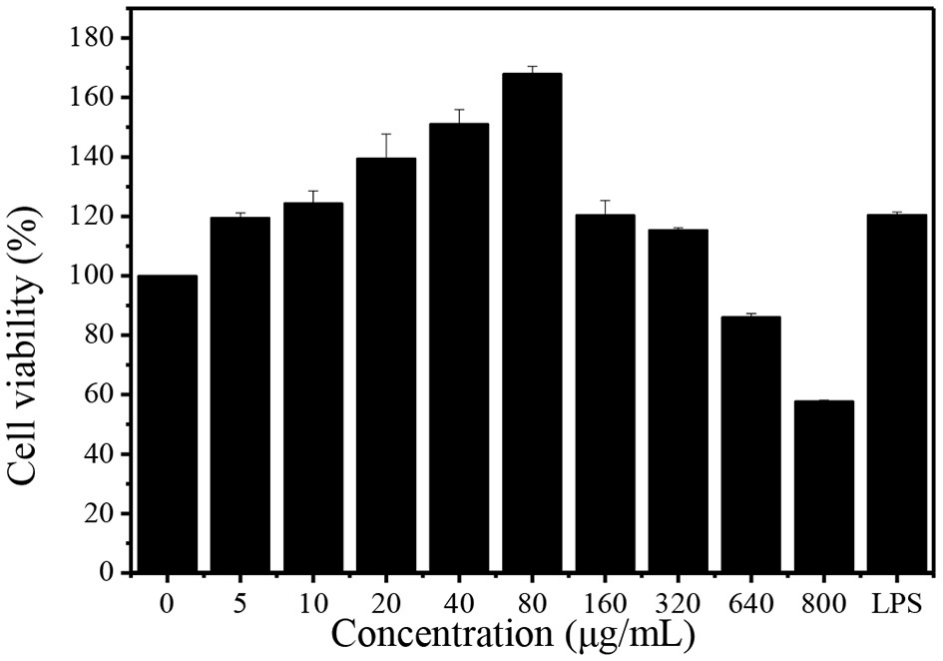
Effect of DHPs-1 on the viability of RAW264.7 cells.

Macrophages play an important role in the immune response to defend pathogens and retain homeostasis. Phagocytosis is a fundamental cellular process which plays a significant role in the immune system (17). The effect of DHPs-1 on the phagocytic activity of RAW264.7 cells was detected using CCK-8 method. The result indicated that the DHPs-1 displayed no toxicity against RAW264.7 cells with concentrations increasing in the range of 5–320 μg/mL, and all the cells were survived in this concentration range (Figure 6). At the concentration of 80 μg/mL, DHPs-1 presented the greatest pro-proliferative activity (68%) as compared with the control group. When the concentration reached 160 μg/mL, the promoting rate was declined compared with the concentration group of 80 μg/mL, which demonstrated that DHPs-1 significantly promoted the proliferation of RAW264.7 cells at lower concentration. However, DHPs-1 indicated inhibitory property of RAW264.7 cells at 640 μg/mL (14%) and at 800 μg/mL (42%). These results of macrophages proliferation were compared with previous studies, showing that DHPs-1 has a higher rate of macrophages proliferation (29). Above all, DHPs-1 could have an immune effect *via* regulation of RAW264.7 cell growth (30).

#### Q-PCR Test Result of Cytokines

**Figure 7:**
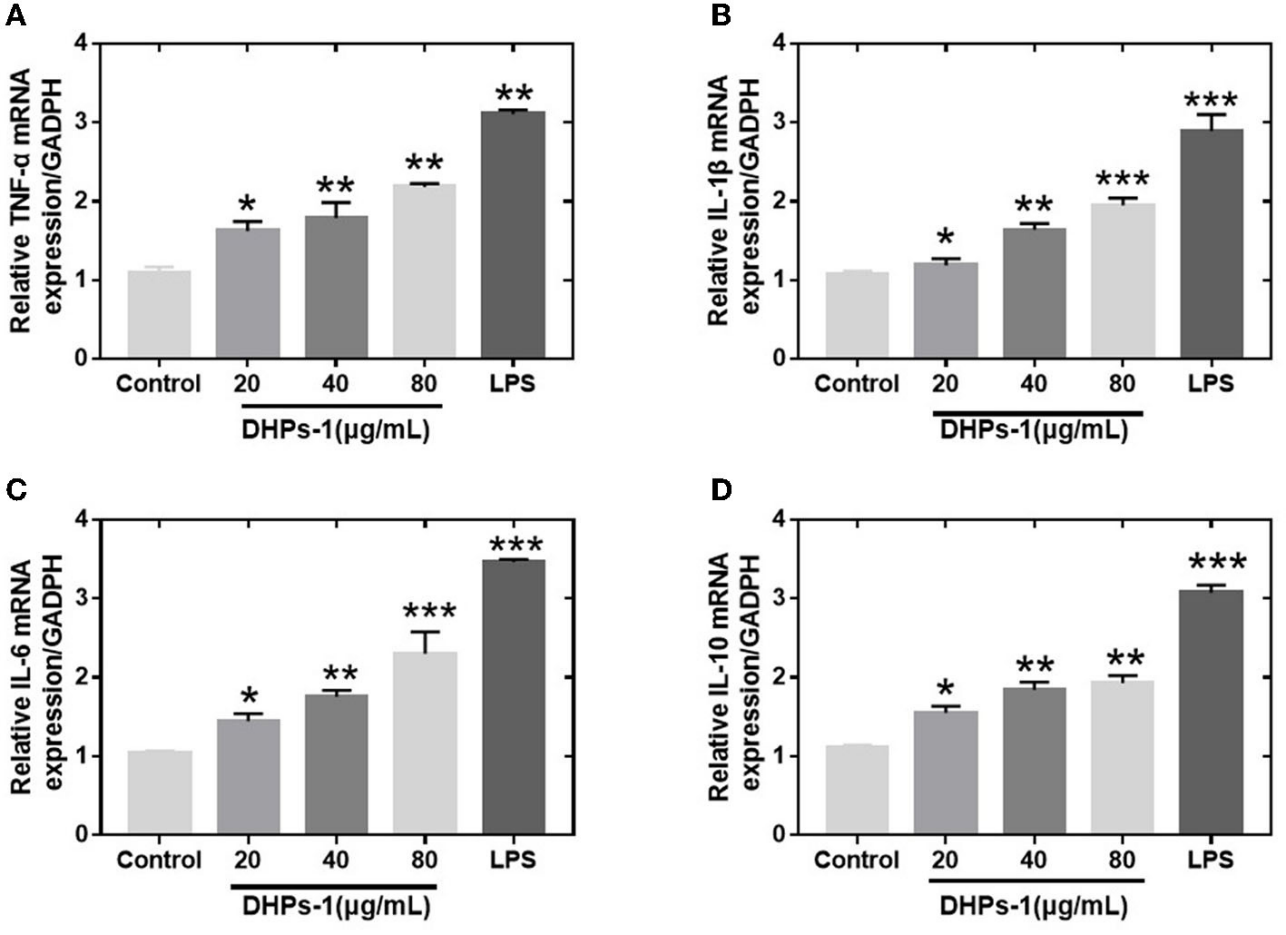
Effects of DHPs-1 on the expression of cytokine genes in RAW264.7 cells **(A–D)**: Expression of TNF-α **(A)**, IL-1β **(B)**, IL-6 **(C)**, and IL-10 **(D)** mRNA in RAW264.7 cells treated with DHPs-1 or LPS groups for 12 h. Significant differences from control group are denoted by *p < 005, **p < 0.01, ***p < 0.001.

The proliferation of tumor cells can be inhibited by activated macrophages and released TNF-α. The cytokines (IL-1β, IL-6, and TNF-α) have been regarded as major immune mediators and they could be produced by activating immune cells (T-cells, B-cells, and macrophages). However, excessive secretion of these cytokines could harmful and affect the repair process of damaged cells and tissues. Therefore, anti-inflammation cytokine (IL-10) is used to prevent the harmful effects of excessive macrophage activation (31). Transcript levels of the above four cytokine genes were identified by Q-PCR. The mRNA transcriptions of TNF-α, IL-1β, IL-6, IL-10 genes induced by DPHs-1 or LPS groups were significantly improved compared with the control group (Figure 7). The data suggested that DHPs-1 promoted the release of TNF-α, IL-1, IL-6, IL-10 through up-regulating the mRNA expression of TNF-α, IL-1, IL-6, IL-10. The above results indicate that DPHs-1 is a potential immunomodulatory agent and antioxidant agent. In this work, DHPs-1 from *D. huoshanense* was rich in β-1,4-d-Glc*p*, which might contribute to its antioxidant and immunological activity. This result is in accordance with that of the polysaccharide from the wild mushroom Paxillus involutus (32). Further research will be performed to investigate the relationship among the monosaccharide composition, molecular weight, crystallinity degree, spatial conformation and bioactivity (33).

## Conclusion

A novel polysaccharide DHPs-1 with a weight-average molecular weight of 50 KDa was extracted and purified from *Dendrobium huoshanense* by DEAE-cellulose and Sephadex G-100 chromatography. Its structure had a backbone of 1,4-linked β-Glc*p*, and 1,4,6-linked β-Gal*p*. The 1,3,4-linked Gal*p*, 1,4-linked Man*p*, 1,4-Gal*p*, and 1,3,4,6-Gal*p* could be in the backbone or the side chains. The non-reducing terminal was α-Man*p*. SEM revealed that the DHPs-1 had an irregular, fibrous, filamentous surface. XRD analysis revealed it had both crystalline and amorphous structural region. DHPs-1 displayed favorable thermal stability. We successfully evaluated its antioxidant activities (DPPH• and •OH scavenging activities) and immunomodulatory activities in RAW264.7 macrophage cells. DHPs-1 was found to possess strong DPPH• scavenging activity and significant immunological activities. These results suggest that it could potentially be used as a natural antioxidant and immunological agent in functional foods or medicines. Further experiments need to be performed assess its bioactivities to determine the details of the mechanisms of these activities.

## Data Availability Statement

The datasets presented in this study can be found in online repositories. The names of the repository/repositories and accession number(s) can be found in the article/Supplementary Material.

## Author Contributions

LW, Y-gM, B-jM, L-pG, X-lY, and XZ contributed to conception and design of the study. NL, C-fN, and X-xL organized the database. Y-gM, NL, C-fN, and X-xL performed the statistical analysis. LW, B-jM, and L-pG wrote the first draft of the manuscript. B-jM, L-pG, and X-lY wrote sections of the manuscript. All authors contributed to manuscript revision, read, and approved the submitted version.

## Funding

This work was financially supported by the National Natural Science Foundation of China (Nos. 81872755 and 82073714), the National Key R&D Program of China (2020YFC1712703), and the National Civil Affairs Commission's Young and Middle-Aged Talents Training Program (MZR20008).

## Conflict of Interest

The authors declare that the research was conducted in the absence of any commercial or financial relationships that could be construed as a potential conflict of interest.

## Publisher's Note

All claims expressed in this article are solely those of the authors and do not necessarily represent those of their affiliated organizations, or those of the publisher, the editors and the reviewers. Any product that may be evaluated in this article, or claim that may be made by its manufacturer, is not guaranteed or endorsed by the publisher.
