# Supplementary material for: Structure and Bioactivities of a Novel Polysaccharide Extracted From Dendrobium huoshanense by Subcritical Water
Source: Front Nutr. 2022 Apr 26;9:877871. doi: 10.3389/fnut.2022.877871 (PMC9087634; doi:10.3389/fnut.2022.877871)
Supplement: Supplementary file 1 [file Data_Sheet_1.PDF]

Structure and bioactivities of a novel polysaccharide extracted from *Dendrobium* huoshanense by subcritical water

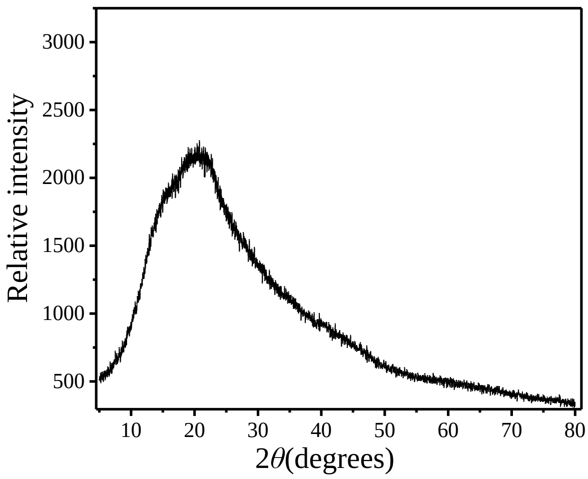

Fig. S1. X-ray diffraction pattern of DHPs-1

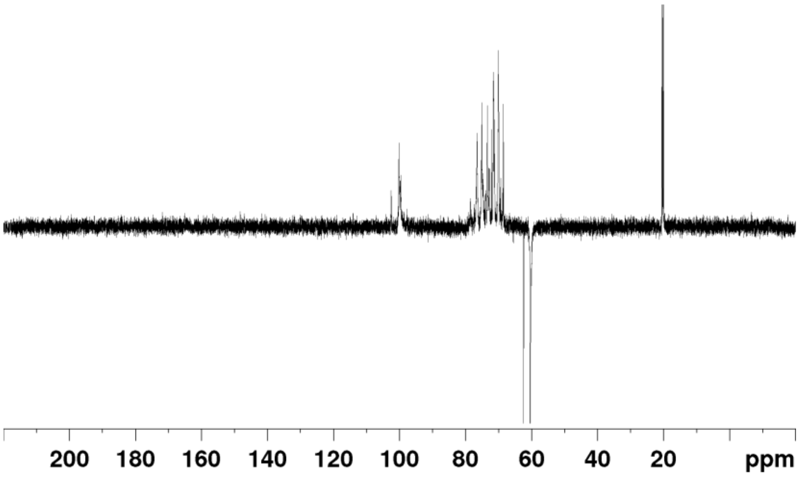

Fig. S2. DEPT-135 spectrum of DHPs-1
